# Gemcitabine and oxaliplatin combination in patients with advanced adrenocortical carcinoma

**DOI:** 10.1210/jendso/bvag152

**Published:** 2026-07-01

**Authors:** Lily Chen, Vania Balderrama-Brondani, Leonardo P Marcal, Camilo Jimenez, Jeena Varghese, Amishi Y Shah, Mouhammed Amir Habra, Matthew T Campbell

**Affiliations:** Department of Internal Medicine, McGovern Medical School, The University of Texas Health Science Center at Houston, Houston, TX 77030, USA; Department of Endocrine Neoplasia and Hormonal Disorders, Division of Internal Medicine, The University of Texas MD Anderson Cancer Center, Houston, TX 77030, USA; Department of Abdominal Imaging, The University of Texas MD Anderson Cancer Center, Houston, TX 77030, USA; Department of Endocrine Neoplasia and Hormonal Disorders, Division of Internal Medicine, The University of Texas MD Anderson Cancer Center, Houston, TX 77030, USA; Department of Endocrine Neoplasia and Hormonal Disorders, Division of Internal Medicine, The University of Texas MD Anderson Cancer Center, Houston, TX 77030, USA; Department of Genitourinary Medical Oncology, Division of Cancer Medicine, The University of Texas MD Anderson Cancer Center, Houston, TX 77030, USA; Department of Endocrine Neoplasia and Hormonal Disorders, Division of Internal Medicine, The University of Texas MD Anderson Cancer Center, Houston, TX 77030, USA; Department of Genitourinary Medical Oncology, Division of Cancer Medicine, The University of Texas MD Anderson Cancer Center, Houston, TX 77030, USA

**Keywords:** adrenocortical carcinoma, gemcitabine, oxaliplatin, chemotherapy

## Abstract

**Context:**

Adrenocortical carcinoma (ACC) is a rare, aggressive malignancy with limited treatment options beyond first-line therapy, underscoring the need for effective salvage regimens.

**Objective:**

To evaluate the clinical activity and safety of gemcitabine and oxaliplatin (GemOx) in advanced ACC.

**Design:**

Retrospective cohort study conducted from April 2023 to April 2024 with longitudinal follow-up.

**Setting:**

Single-center tertiary referral cancer center.

**Patients or Other Participants:**

Fourteen patients with histologically confirmed advanced ACC treated with GemOx were included. Patients were heavily pretreated, with a median of 3 prior systemic therapies (range, 2-9); 43% had hormonally functional tumors.

**Intervention(s):**

Gemcitabine (1000 mg/m^2^ on days 1 and 8) and oxaliplatin (130 mg/m^2^ on day 1) every 3 weeks until disease progression or unacceptable toxicity.

**Main Outcome Measure(s):**

Primary outcomes were progression-free survival (PFS) and overall survival (OS). Secondary outcomes included objective response rate per Response Evaluation Criteria In Solid Tumors 1.1 and treatment-related toxic effects.

**Results:**

After a median follow-up of 10.7 months (95% CI, 8.5-15.7), median PFS was 3.2 months (95% CI, 0.4-6.0) and OS was 13.0 months (95% CI, 3.6-22.5). Among 13 evaluable patients, 2 (15.4%) achieved partial response, 8 (61.5%) had stable disease, and 3 (23.1%) had progressive disease, yielding a disease control rate of 76.9%. No treatment-related deaths occurred.

**Conclusion:**

GemOx demonstrated modest clinical activity with manageable safety in heavily pretreated patients with advanced ACC. These findings suggest a potential role for GemOx as a salvage option, though validation in larger prospective studies is needed.

Adrenocortical carcinoma (ACC) is a rare and aggressive malignancy with limited treatment options and poor overall prognosis. The stage at diagnosis remains the most important prognostic factor, with a 5-year survival rate of <15% in patients with metastatic disease [1, 2]. For advanced ACC, mitotane remains the backbone of systemic therapy, used alone or in combination with cytotoxic agents. The FIRM-ACT trial established the combination of etoposide, doxorubicin, and cisplatin plus mitotane (EDP-M) as the standard first-line regimen, with a median progression-free survival (PFS) of 5.1 months [3]. However, EDP-M is associated with substantial toxicity and limited durability of response. Beyond progression on platinum-based chemotherapy, systemic options remain sparse, and no second-line standard of care has been defined, underscoring the urgent need for more effective therapies in this setting.

Second-line treatment options are informed primarily by small phase II trials and retrospective series. Gemcitabine-based regimens have emerged as potential alternatives. A phase II study evaluating gemcitabine with fluoropyrimidines (capecitabine or 5-fluorouracil) in 28 heavily pretreated ACC patients reported a disease control rate of 46% at 4 months and a median time to progression of 5.3 months [4]. A subsequent retrospective multicenter study involving 145 patients treated with gemcitabine-based chemotherapy, primarily with concomitant capecitabine, observed a median PFS of 12 weeks, an objective response rate of 4.9%, and grade 3 or 4 toxic effects in 11% of cases [5]. These findings reflect the limited efficacy of current second-line strategies.

Several molecularly targeted agents have been investigated following progression of ACC after mitotane or EDP-M [6]. To date, none of these agents have been approved for use in advanced ACC. The identification of predictive biomarkers to determine which tumors will respond to specific treatments, including immunotherapies, remains an evolving field. In the absence of reliable predictive biomarkers, optimizing treatment strategies beyond first-line therapy remains a critical unmet need in ACC management.

Gemcitabine–oxaliplatin (GemOx) is a chemotherapeutic regimen used in various solid tumors, demonstrating efficacy in pancreatic, biliary tract, and ovarian cancers [7]. Its mechanism combines the nucleoside analog gemcitabine with the platinum-based agent oxaliplatin, offering a synergistic cytotoxic effect [8]. In pancreatic cancer, GemOx has been shown to improve disease control and symptom relief in advanced-stage disease; while in biliary tract malignancies, it has demonstrated activity comparable to that of cisplatin-based regimens [9, 10]. Notably, GemOx has also been explored in sarcomas, germ cell tumors, and neuroendocrine tumors, supporting its potential application in rare and aggressive malignancies [11-14].

Given the limited options after EDP-M, this study aimed to evaluate the efficacy and safety of GemOx in a cohort of patients with advanced ACC.

## Materials and methods

### Study design, patient selection, and treatment

This retrospective case series included 14 patients with advanced ACC treated at The University of Texas MD Anderson Cancer Center who received GemOx during April 2023 through April 2024. The study protocol (PA 12-0933) was approved by the institutional review board. Eligible patients met the following inclusion criteria: histologically confirmed ACC; radiological, clinical, and biochemical evidence of progressive disease after first-line therapy; ineligibility for loco-regional therapies; measurable disease per Response Evaluation Criteria In Solid Tumors (RECIST; version 1.1); life expectancy of ≥3 months as estimated by the oncologist; age ≥18 years; Eastern Cooperative Oncology Group (ECOG) performance status 0-2; adequate organ function; and ability to provide informed consent. The primary analysis consisted of PFS and overall survival (OS). The secondary analysis included best overall objective response and treatment-related toxicity.

Clinical and pathologic characteristics were determined both at initial ACC diagnosis and shortly before GemOx initiation. These variables included sex, age, ECOG performance status, tumor stage (per European Network for the Study of Adrenal Tumors classification), tumor size, tumor hormonal activity, Weiss score, Ki-67 proliferation index, metastatic sites and number of involved organs, and number of prior therapy lines (Table 1). Follow-up data during GemOx treatment were collected from the patients' medical records and are further described below.

**Table 1:** Patients' clinical and pathological characteristics at initial diagnosis and at the start of gemcitabine and oxaliplatin combination chemotherapy

Variables	*N* (%)^a^
Sex	
Female	5 (36%)
Male	9 (64%)
Age (years), median (range)	53.7 (29.5-68.5)
Racial/ethnic group	
Asian/Pacific Islander	2 (14%)
Hispanic	1 (7%)
Non-Hispanic White	11 (79%)
Time from initial ACC diagnosis (months), median (range)	41.1 (9.8-229.9)
Hormonally active tumor	
Androgen-producing	1 (7%)
Cortisol-producing (including mixed hormonal production)	5 (36%)
Nonfunctioning	8 (57%)
Number of metastatic sites at GemOx start	
1	1 (7%)
2	7 (50%)
3	5 (36%)
4	1 (7%)
Sites of metastases	
Lung	10 (71%)
Liver	13 (93%)
Abdomen/peritoneum	6 (43%)
Bone	4 (29%)
Others	1 (7%)
ECOG performance status at GemOx start	
0	5 (36%)
1	9 (64%)
≥2	0
Pathology	
Ki67 (%), median (range)^b^	26.5 (4-58)
Weiss score, median (range)^b^	4 (2-8)
History of adrenal surgery	
Yes	14 (100%)
Systemic therapies before GemOx	
Platinum-based chemotherapy	14 (100%)
Mitotane	13 (93%)
Immunotherapy	13 (93%)
TKI	9 (64%)
Other	3 (21%)
Number of lines of prior systemic therapy	
1-2	1 (7%)
3-4	9 (64%)
≥5	4 (29%)

Abbreviations: ACC, adrenocortical carcinoma; ECOG, Eastern Cooperative Oncology Group; GemOx, gemcitabine and oxaliplatin combination.

^a^Data are presented as number (%) unless otherwise indicated.

^b^Ten of the 14 patients had this information available.

Patients received gemcitabine (1000 mg/m^2^ intravenously on days 1 and 8) and oxaliplatin (130 mg/m^2^ intravenously on day 1) every 3 weeks. Two patients also received concurrent mitotane therapy with the aim of reaching a plasma concentration of 14-20 mg/L; however, neither reached the therapeutic threshold (≥14 mg/L). Chemotherapy continued until disease progression or unacceptable toxicity. Dose modifications and discontinuation of individual agents were performed at the clinician's discretion.

### Evaluation of response and toxicity

Baseline evaluation included physical examination, comprehensive biochemical testing (including routine laboratory panels and steroid hormone levels), and tumor assessment by imaging. Imaging modalities consisted primarily of whole-body computed tomography and/or fluorodeoxyglucose positron emission tomography (PET/CT).

Of the 14 patients, 13 completed standardized follow-up evaluations; 1 patient was lost to follow-up. Follow-up imaging was typically performed every 8-12 weeks to assess disease status. Tumor response was independently evaluated by a board-certified radiologist using RECIST version 1.1 criteria based on changes in target lesion size. Responses were categorized as complete response (CR), partial response (PR), stable disease (SD), or progressive disease (PD). Objective response rate (ORR) was defined as CR or PR; clinical benefit rate was defined as CR, PR, or SD sustained for at least 4 months.

Adverse events were recorded during follow-up visits and summarized retrospectively from medical records. Toxic effects were graded according to the Common Terminology Criteria for Adverse Events (CTCAE; version 5.0).

### Statistical analysis

Descriptive statistics were used to analyze clinical parameters. Continuous variables were categorized using optimal cutoff values. PFS was defined as the time from the initiation of GemOx to the first radiological evidence of disease progression or death, and OS was calculated from the time of initiation of chemotherapy, both using the Kaplan–Meier technique. Both survival measures were censored at last follow-up. Median follow-up time was calculated using the reverse Kaplan–Meier technique. All statistical analyses were conducted using SPSS Statistics software, version 23.0 (IBM).

## Results

### Patients' clinicopathological characteristics, treatment, and toxicity

Fourteen patients with advanced ACC were treated with GemOx chemotherapy. Baseline clinical and pathological characteristics are summarized in Table 1. The median age at GemOx initiation was 54 years (range, 30-69), and 9 patients (64%) were male. All patients had metastatic disease at GemOx initiation, and 6 patients (43%) had evidence of hormonal hypersecretion.

All patients had undergone prior unilateral adrenalectomy. Thirteen patients (92.9%) required glucocorticoid replacement therapy with hydrocortisone or dexamethasone for adrenal insufficiency. One patient had an androgen-producing tumor and did not receive antiandrogen therapy.

The median Ki-67 proliferation index was 26.5% (range, 4-58). Thirteen patients (93%) had received prior adjuvant mitotane therapy, and mitotane plasma levels were available for 12 patients; of these, 8 (67%) had subtherapeutic mitotane concentrations (<14 mg/L) at the time of GemOx initiation.

The median number of metastatic sites was 2 (range, 1-4). Liver metastases were present in 13 patients (93%), lung metastases in 10 (71%), and extrahepatic abdominal or peritoneal involvement in 6 (43%). Among patients with liver involvement, 1 underwent hepatic embolization and 2 received radiofrequency ablation prior to GemOx. The median time from initial diagnosis to GemOx initiation was 41.2 months. At baseline, 9 patients (64%) had an ECOG performance status ≥1 and reported disease-related symptoms.

All patients had received at least 2 prior systemic therapies for advanced disease. Prior systemic therapies were discontinued due to disease progression. GemOx was administered as third-line therapy in 1 patient (7%), fourth-line in 8 patients (57%), and fifth- to tenth-line in 5 patients (36%). Prior treatments included mitotane, platinum-based chemotherapy, streptozocin, immunotherapy, and tyrosine kinase inhibitors (TKIs). Three patients had also participated in early phase clinical trials evaluating investigational agents, including ATR-101 (ACAT-1 inhibitor), AZD1775 (Wee1 inhibitor), pembrolizumab in combination with a LAG-3 inhibitor, EO2401 (a microbiome-derived vaccine), an SHP2 inhibitor, an NKG2A antagonist, and IO-202 in combination with pembrolizumab. All patients received both gemcitabine and oxaliplatin, with a median of 3 cycles administered (range, 1-10).

Treatment was generally well tolerated. Grade 1-2 adverse events occurred in 6 patients (50%) and included fatigue, nausea or vomiting, diarrhea, anemia, thrombocytopenia, renal insufficiency, peripheral neuropathy, and oxaliplatin-associated cold sensitivity. One patient (8.3%) experienced a grade 4 adverse event consisting of upper gastrointestinal bleeding due to a 20-mm duodenal ulcer requiring hospitalization and transfusion; hemostasis was achieved with endoscopic intervention, and hemoglobin subsequently stabilized. Toxicities are summarized in Table S1 [15].

### Response to therapy and survival analysis

**Figure 1:**
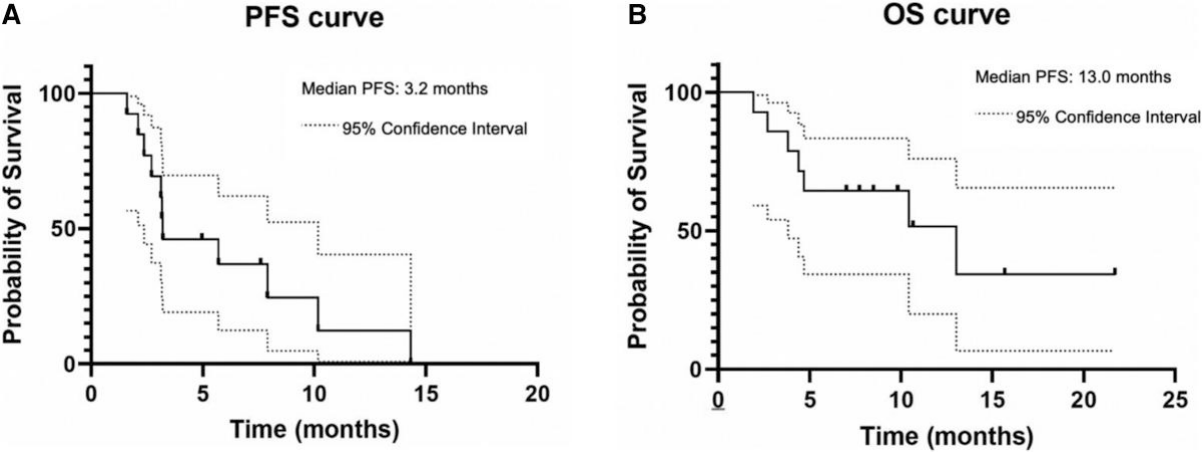
Kaplan–Meier curves showing progression-free survival (A) and overall survival (B) for the entire cohort (*n* = 14). Solid lines represent survival probabilities.

Of the 14 patients enrolled, 13 were eligible for response and survival analysis; 1 patient was lost to follow-up. The median follow-up was 10.7 months (95% CI, 8.5-15.7). At the time of analysis, with the last update in February 2025, the median PFS was 3.2 months (95% CI, 0.4-6.0) (Fig. 1A), and the median OS was 13.0 months (95% CI, 3.6-22.5) (Fig. 1B). PFS rates at 6 and 12 months were 44.4% and 12.5%, respectively.

During the follow-up period, 7 patients died, all from progressive ACC. One patient died from ACC before receiving the second cycle of GemOx (81 days after cycle 1); the death was deemed unrelated to treatment and due to disease progression.

**Figure 2:**
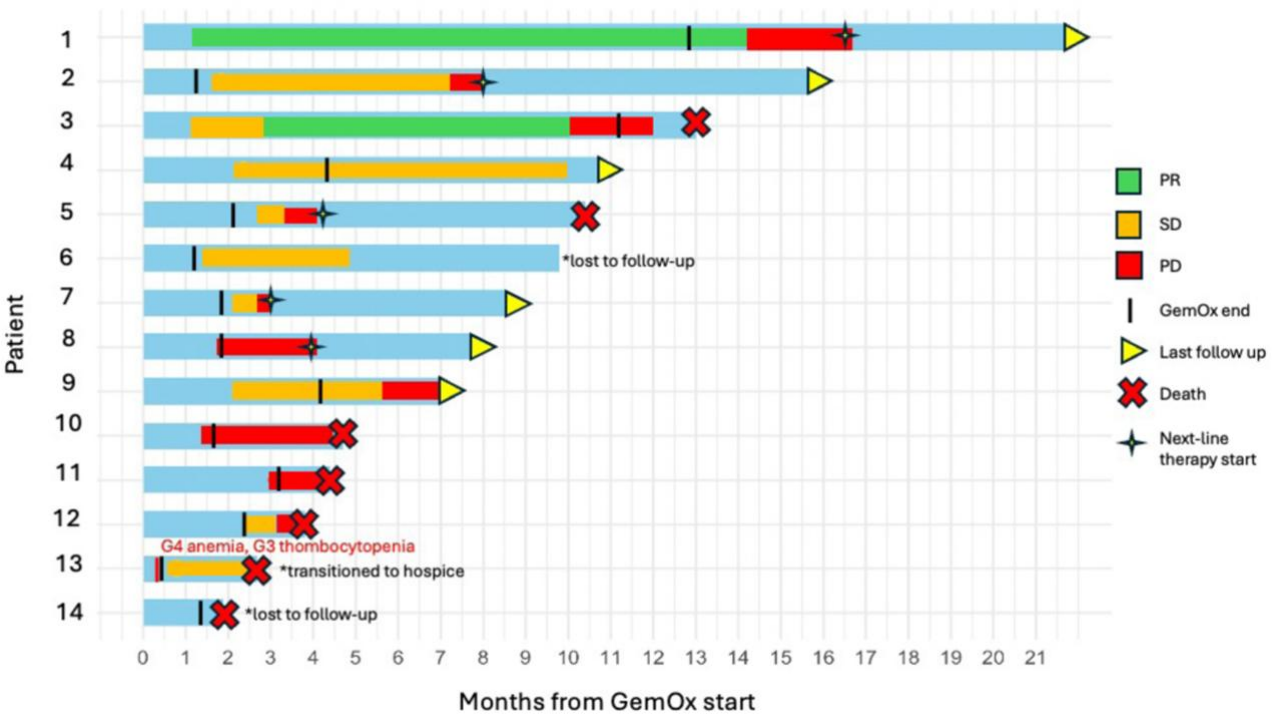
Swimmer's plot illustrating clinical treatment response and therapy duration. Abbreviations: GemOx: gemcitabine and oxaliplatin; PD: progressive disease; PR: partial response; SD: stable disease. Treatment-related toxicities and subsequent therapies are summarized in the Table S1 [15].

**Figure 3:**
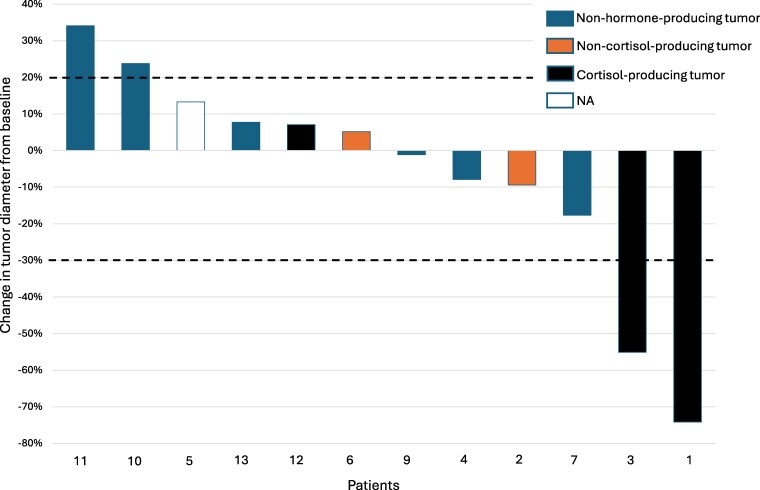
Waterfall plot showing the best overall response in 12 patients with available follow-up imaging, assessed using Response Evaluation Criteria In Solid Tumors (RECIST) version 1.1 based on changes in target lesion size. The dotted lines indicate a 30% reduction and 20% increase in tumor size from the baseline, which are the cutoff points that determine partial response and progressive disease, respectively. Abbreviation: NA, not applicable.

**Figure 4:**
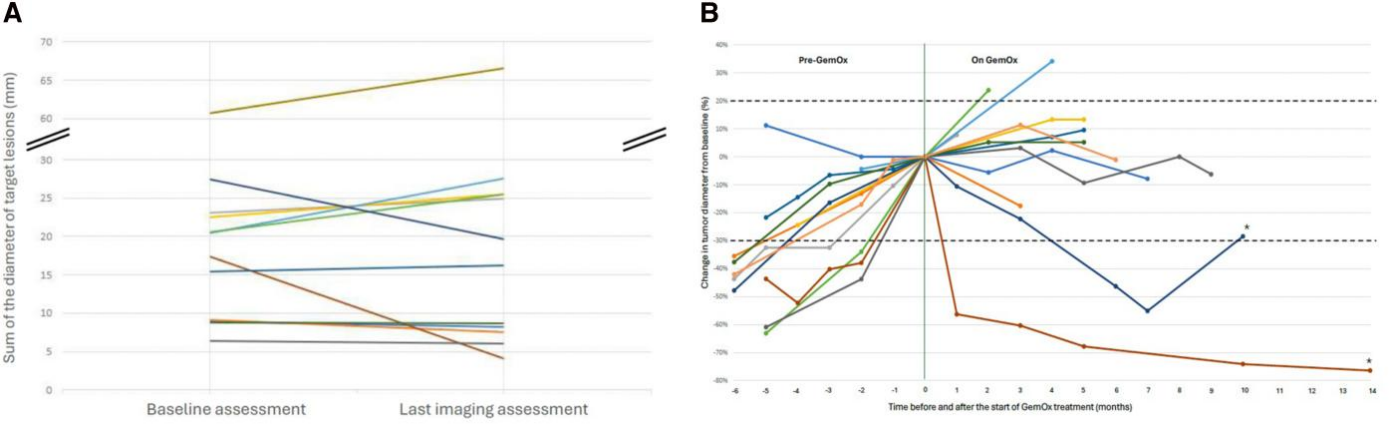
Tumor responses to gemcitabine and oxaliplatin chemotherapy in patients with adrenocortical carcinoma. (A) Spaghetti plot showing the change in total diameter of target lesions from baseline (pretreatment) to the last available imaging assessment during treatment. (B) Spider plot illustrating individual changes in tumor burden over time relative to baseline. Asterisks (*) indicate patients with progressive disease based on nontarget lesions.

Among the 13 evaluable patients, 2 patients (15.4%) had a PR, 8 (61.5%) had SD, and 3 (23.1%) had PD. No CR was observed. The ORR was 15.4% and the disease control rate (DCR, defined as PR or SD) was 76.9% (Table 2 and Fig. 2). Notably, both patients who achieved a PR had cortisol-secreting tumors (Fig. 3). Longitudinal changes in total diameter of target lesions and individual changes in tumor burden over time relative to baseline are shown in Fig. 4A and 4B, respectively. One patient experienced PD at 1.87 months based on clinical progression; however, radiographic measurements were not available for this patient. At 4 months following initiation of GemOx, 46.2% of patients demonstrated clinical benefit, defined as PR or SD. Following disease progression, 5 patients (35.7%) received subsequent systemic therapy, including TKI monotherapy (*n* = 1), combination immunotherapy (*n* = 1), TKI plus immunotherapy (*n* = 2), and chemotherapy (*n* = 1) (Table S1 [15]).

**Table 2:** Activity and adverse events of gemcitabine and oxaliplatin combination chemotherapy

Endpoints	Value
Duration of GemOx therapy (months), median (range)	3.58 (0.8-12.9)
Progression-free survival (months), median (range)	3.2 (0.4-6.0)
Best objective response to therapy^a^	
Partial response, *n* (%)	2 (15.4%)
Stable disease, *n* (%)	8 (61.5%)
Progressive disease, *n* (%)	3 (23.1%)
Adverse events^b^	
Grade 1-2, *n* (%)	6 (50.0%)
Grade 3-4, *n* (%)	1 (8.3%)

^a^Thirteen of the 14 patients were evaluable for response.

^b^Two of the 14 patients were lost to follow-up.

Two patients with metastatic ACC experienced durable responses following treatment with GemOx (Fig. 2, Patients #1 and #3). The first patient had a PR after 2 cycles. Treatment was well tolerated, with only mild neutropenia and peripheral neuropathy. After completing 4 cycles, she remained in PR off therapy. One year later, disease progression in the liver prompted GemOx reinitiation. Although therapy was generally tolerated, she developed an oxaliplatin-induced hypersensitivity reaction characterized by transient slurred speech, which resolved the same day. Two months later, PET/CT revealed progression of a solitary hepatic lesion, confirmed as metastatic ACC on biopsy and later complicated by subcapsular hemorrhage. Following clinical stabilization, she was transitioned to lenvatinib plus pembrolizumab.

The second patient initially tolerated GemOx well. After 5 cycles, treatment was held because of worsening fatigue and nausea, along with grade 2 peripheral neuropathy. He remained in PR for 7 months. At 10 months, he developed disease progression, prompting GemOx reinitiation. Despite therapy, imaging demonstrated extensive progression, including splenic involvement, tumor thrombus, and new intra-abdominal metastases. He was subsequently transitioned to hospice care.

## Discussion

In this retrospective cohort, GemOx demonstrated limited but potentially relevant activity in patients with metastatic ACC, a population with few effective second-line options. The median PFS of 3.2 months underscores the challenges of disease control in this setting, particularly in heavily pretreated patients with aggressive disease biology. Although short, this PFS falls within the range reported for other systemic regimens, suggesting that GemOx may provide transient disease control for selected patients. In this context, the clinical utility of GemOx should be considered in terms of disease stabilization and tolerability rather than durable responses.

Disease stabilization was observed in a subset of patients, with a 4-month clinical benefit rate of 46%, supporting its role as a cytotoxic option when treatment goals include symptom control and delaying progression. These findings are consistent with prior studies of gemcitabine-based regimens. A prospective phase II trial of gemcitabine with fluoropyrimidines (capecitabine or 5-fluorouracil) reported a DCR of 46% [4], while another study of gemcitabine—capecitabine in 50 patients demonstrated a 30% clinical benefit rate at 4 months and a median PFS of 3.0 months [16]. Similarly, carboplatin and etoposide yielded a higher PR rate (33%) but comparable DCR (66%) in a small retrospective series [17]. Temozolomide, an oral alkylating agent, achieved a DCR of 35.8%, median PFS of 3.5 months, and OS of 7.2 months in 28 patients [18]. Collectively, these comparisons suggest that GemOx provides efficacy broadly comparable to other cytotoxic options, with a numerically higher disease control rate and similar short PFS, reinforcing that stabilization rather than tumor shrinkage is the predominant therapeutic benefit across regimens in this space.

Importantly, the relatively favorable OS of 13.0 months in our cohort, despite a short PFS, may reflect both patient selection and the ability to maintain therapy due to tolerability. This highlights a key aspect of clinical utility in this setting: regimens that are well tolerated may allow sustained treatment exposure and facilitate subsequent lines of therapy, which can meaningfully impact OS even when initial disease control is limited in duration.

Interestingly, both patients who achieved PRs had cortisol-secreting tumors, a subgroup typically associated with poor prognosis and treatment resistance [19]. While limited by small numbers, this observation raises the possibility that GemOx may retain activity in biologically aggressive subtypes and supports further evaluation in molecularly or clinically stratified cohorts.

GemOx was generally well tolerated, with only 1 patient (8%) experiencing a grade ≥3 treatment-related adverse event and most toxicities limited to grade 1-2. This compares favorably with gemcitabine—capecitabine regimens, which report grade ≥3 toxicities in ∼11% of patients [5]. This favorable safety profile is particularly relevant when compared with targeted therapies, where toxicity can limit treatment duration. Thus, beyond efficacy, tolerability represents a meaningful component of GemOx's clinical utility, especially for patients with poor performance status or limited reserve.

The modest PFS observed with GemOx is also consistent with outcomes reported for immunotherapy-based approaches in metastatic ACC. In the JAVELIN trial, avelumab achieved a median PFS of 2.6 months and OS of 10.6 months in heavily pretreated patients [20]. Pembrolizumab monotherapy demonstrated ORRs of 14%–23%, but median PFS remained short at approximately 2.1 months [21, 22]. Combination therapy with lenvatinib and pembrolizumab improved median PFS to 5.5 months in a small cohort [23], though with increased complexity and potential toxicity. Within this context, GemOx achieved an ORR of 15%, median PFS of 3.2 months, and OS of 13.0 months, suggesting comparable efficacy to immunotherapy-based regimens, with a distinct mechanism and favorable tolerability profile. These comparisons reinforce that across available therapies, durable responses remain uncommon, and disease stabilization is a clinically meaningful endpoint.

Cabozantinib monotherapy has demonstrated a longer median PFS of 6.0 months in a small cohort, but at the cost of substantial toxicity, with grade ≥3 adverse events reported in 61% of patients [24]. In contrast, GemOx was associated with largely low grade toxicities reflecting the known safety profile of this combination and the retrospective nature of data collection which often reports less toxicity than a prospective trial. The toxicity burden between a TKI and chemotherapy are different and treatment selection should be informed by both tolerability and patient fitness. Accordingly, GemOx may be particularly relevant for patients who are not candidates for or are intolerant of targeted therapies, or when a cytotoxic approach is preferred to achieve symptom control and short-term disease stabilization.

From a biologic perspective, tumor epigenetics may influence responsiveness to gemcitabine-based therapy. ACC subtypes characterized by high levels of DNA methylation (CIMP-high) are associated with worse prognosis [25], and preclinical data suggest that demethylating strategies may enhance sensitivity to gemcitabine [26, 27]. These observations support the integration of molecular profiling in future studies to better define patient subtypes most likely to benefit from GemOx.

This study has several limitations, including its retrospective design, small sample size, lack of a comparator arm, and heterogeneity in prior therapies and mitotane exposure. These factors limit definitive conclusions regarding comparative efficacy. Nonetheless, our findings provide real-world evidence that GemOx is well tolerated and can achieve disease control in a subset of patients with metastatic ACC, even in the context of a short median PFS. In the absence of a clearly established second-line regimen, GemOx represents a reasonable salvage option for carefully selected patients. Prospective studies are warranted to better define its role and to identify predictive biomarkers that may guide patient selection.

## Conclusion

This retrospective analysis suggests that GemOx is a feasible salvage regimen with limited but potentially meaningful activity in patients with metastatic ACC who have exhausted standard therapies. In this heavily pretreated cohort, GemOx achieved a 4-month clinical benefit rate of 46%, median PFS of 3.2 months, and OS of 13.0 months. While the PFS was modest, outcomes were comparable to those reported with other systemic regimens in this setting, and the regimen was generally well tolerated, with a low incidence of grade ≥3 toxicities consistent with prior gemcitabine-based studies.

While the study is limited by its retrospective design and sample size, these findings add to the limited evidence base on systemic options for metastatic ACC. Future prospective trials are warranted to clarify the role of GemOx, ideally integrating biomarker-driven approaches such as DNA hypermethylation status and other molecular features to guide personalized treatment strategies in this rare and aggressive malignancy.

## Data Availability

Original data generated and analyzed during this study are included in this published article or in the data repositories listed in References.
